# Mobile phone tracking: in support of modelling traffic-related air pollution contribution to individual exposure and its implications for public health impact assessment

**DOI:** 10.1186/1476-069X-12-93

**Published:** 2013-11-04

**Authors:** Hai-Ying Liu, Erik Skjetne, Mike Kobernus

**Affiliations:** 1 NILU - Norwegian Institute for Air Research, Instituttveien 18, Kjeller 2027, Norway; 2 Statoil Research Center, Arkitekt Ebbells Veg 10, Rotvoll, Trondheim 7005, Norway

**Keywords:** Citizen participation, Health impact assessment, Individual exposure, Mobile phone tracking, Public health, Social network, Traffic-related air pollution

## Abstract

We propose a new approach to assess the impact of traffic-related air pollution on public health by mapping personal trajectories using mobile phone tracking technology in an urban environment. Although this approach is not based on any empirical studies, we believe that this method has great potential and deserves serious attention. Mobile phone tracking technology makes it feasible to generate millions of personal trajectories and thereby cover a large fraction of an urban population. Through analysis, personal trajectories are not only associated to persons, but it can also be associated with vehicles, vehicle type, vehicle speed, vehicle emission rates, and sources of vehicle emissions. Pollution levels can be estimated by dispersion models from calculated traffic emissions. Traffic pollution exposure to individuals can be estimated based on the exposure along the individual human trajectories in the estimated pollution concentration fields by utilizing modelling tools. By data integration, one may identify trajectory patterns of particularly exposed human groups. The approach of personal trajectories may open a new paradigm in understanding urban dynamics and new perspectives in population-wide empirical public health research. This new approach can be further applied to individual commuter route planning, land use planning, urban traffic network planning, and used by authorities to formulate air pollution mitigation policies and regulations.

## Background

According to the United Nations (UN), more than 50% of the world’s population lives in urban areas and this number will increase to 70% by 2050 [1]. Road traffic is the main source of pollution in well-developed urban regions worldwide. Traffic-related air pollution may have diverse impacts on health including exacerbation of asthma, onset of childhood and adult asthma, non-asthma respiratory symptoms, impaired lung function, total and cardiovascular mortality, and cardiovascular morbidity, hospitalizations, various cancers, birth outcomes, diabetes and life expectancy [2], etc. Based on a synthesis of the best available evidence, the HEI (Health Effects Institute) identified that an exposure zone within a range of up to 300 to 500 metres from a major road as the area most highly affected by traffic emissions [2]. According to the United States Environmental Protection Agency (U.S. EPA) [3], more than one-third of Nitrogen Oxides (NOx) and one-quarter of Particulate Matter (PM) emissions in the U.S. are from roadways, and about 45 million Americans live within 100 metres of a road. A study by researchers at the University of Southern California (USC) and the California Air Resources Board found that up to half of Los Angeles’ residents total exposure to ultrafine air pollutants occurs while people are travelling in their vehicles [4]. It has frequently been recognised that car drivers are highly exposed to airborne pollution [5,6], and are at an increased risk of developing several types of cancer [7]. A report by the World Health Organization (WHO) showed that long-term air pollution from traffic in Austria, France and Switzerland triggered 300,000 additional cases of bronchitis in children, 15,000 hospital admissions for heart disease and a further 162,000 asthma attacks in children [8].

Different methods have been used to estimate exposure of participants to urban traffic-related air pollution in previous studies. Exposure of participants is often estimated by the concentration either measured at the monitoring site nearest to the participants’ residential address [9,10], or generated by applying dispersion model [11,12] or Land Use Regression (LUR) techniques to the participants’ residential address [13,14], and by accounting for indicators of traffic and other factors such as geospatial location, meteorology and nearby urbanization. Dispersion models can provide information that satisfies both the spatial coverage and temporal variation, but good performance from dispersion modelling requires good input data. In the approach of LUR models it is relatively simple to interpolate a limited number of measurements to a larger population [15]. However, both approaches do not account for personal activities such as time spent in traffic and activity level which will influence exposure considerably [16].

Methods accounting for personal movement include the Personal Environmental Impact Report (PEIR) [17]. A platform that uses location data sampled from everyday mobile phones to calculate personalized estimates of environmental impact and exposure was presented in 2009 [18]. The authors also proposed using mobile phones with a built-in GPS (Global Positioning System) receiver and an accelerometer for identifying individual transportation modes including whether an individual is stationary, walking, running, biking, or in motorized transport [19,20]. This was further explored in the EU-FP7 project ENVIROFI, where the Personal Environmental Information System (mobile phone based application) provided accurate air quality data for the user’s current location based on the GPS coordinates supplied by the user’s phone [21]. The CitiSense project [22] had a mobile air quality system that enabled users to track their personal air quality exposure [23,24]. The above studies did not target population level exposure. Studies using mobile air pollution sensors are only active on a relatively small number of vehicles for which the estimated pollution levels will be derived. Through this brief review of the health effects of traffic, we identified: (i) exposure to traffic-related emissions may cause major adverse health effects on the population at or near air polluted roadways; (ii) existing exposure models do not accurately characterize the exposure fields; and (iii) these disadvantages in exposure estimation can attenuate health effect estimates. To address these issues, studies are needed to characterize the personal exposure to traffic-related air pollution, and to better understand the linkages between traffic-related air pollution and public health effects.

**Figure 1:**
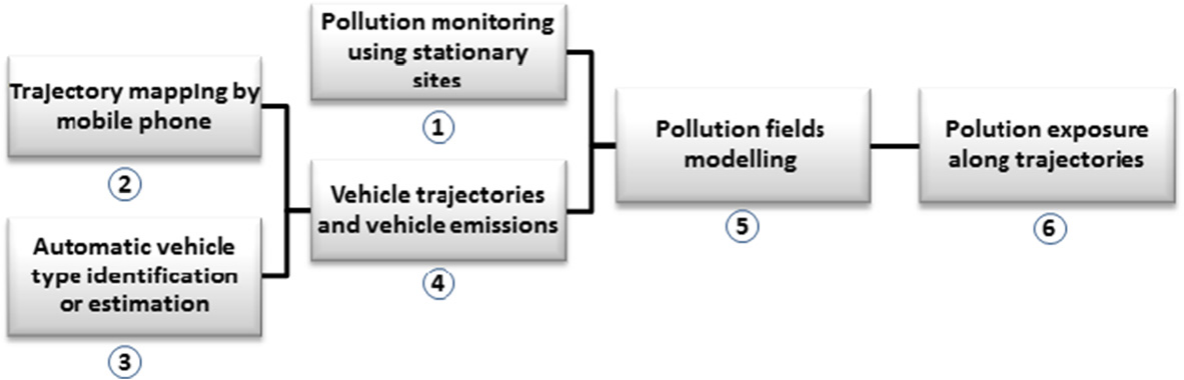
**An overview of work process for modelling traffic-related air pollution contribution to individual exposure by mobile phone tracking.** It is composed of six parts: **①** Pollution monitoring using stationary sites; **②** Trajectory mapping by mobile phone; **③** Automatic vehicle type identification or estimation; **④** Vehicle trajectories and vehicle emissions; **⑤** Pollution field modelling; and **⑥** Pollution exposure along trajectories.

In this study, we propose a conceptual framework to improve exposure assessment by using existing, low cost mobile phone data to obtain individual trajectories to further estimate the concentration of the traffic emission in the roadway network and its contribution to exposure for the urban population. We analyse each element along the work process (Figure 1) from human mobility, through traffic-related air pollution modelling, to exposure and its implications for public health impact assessment. Our proposed methodology is based upon recent advances in social and information networks using Statistical Physics [25] and the techniques of Traffic Engineering in mobile phone networks [26]. The advantage of the proposed study is that we target the entire network of mobile phone users to calculate pollution levels, exposure and health effects. We believe that this has the potential to open up new perspectives in public health research.

The science of individual human trajectories is a new research field based on the recent availability of empirical data on human mobility. Developments in this field have been influenced by results in the multidisciplinary field of network physics [27,28]. Databases and technologies that capture human mobility by mobile phone traces are now available and many interesting features have been discovered including the characteristics of individual human trajectories [29], the interaction between individual mobility and social networks [30], and meso-scopic structure (i.e., the detailed substructures and spatiotemporal flows of mobility) and social aspects of human mobility [31].

Modern network physics is starting to influence public health research. For instance, Health InfoScape, part of the Massachusetts Institute of Technology (MIT) SENSEable Cities and the General Electric Company (GE), aim to create new ways of understanding human health in the U.S. [32]. By analysing data from over 7.2 million anonymous electronic medical records taken from across the U.S., they seek to uncover statistical relationships between space, geography and health.

There is a recent development in Location-Based Services (LBS) [33] through mobile phone technology. Basic mobile phone based positioning systems include: location of nearest base station; trilateration of two or more neighbouring base stations by signal strength or Time Of Arrival (TOA); multilateration (hyperbolic lateration) by Time Difference Of Arrival (TDOA); and network based triangulation by Angle Of Arrival (AOA) using multiple antennas at a base station. More methods are available with third generation mobile systems [34]. Thiragarajan et al. [35-37] used a Hidden Markov Model (HMM) to map noisy phone location data to road segments using mobile phone data from both the GPS and the Global System for Mobile Communications (GSM) systems. The road segments are the hidden states of the system and the noisy data is the input to the algorithm. The mobile phone data are run through the algorithm twice, first to find the mobile phone area sequence and then to find the road segment sequence [38]. Processed position data provided by mobile phones are as accurate as GPS reading intervals of 2 minutes and have much lower energy consumption [39]. According to studies on probabilistic models for mobile phone trajectory estimation from Thiagarajan [39], the misplaced road segment error is approximately only 45 metres. While, on average, mobile phone tracking can retrieve over 75% of a user’s drive accurately, even from highly inaccurate (175 metres raw position error) GSM data.

There are several companies providing individual trajectory mobile phone based traffic monitoring services including: Cellint [40], Intelligent Mechatronic Systems Inc (IMS) [41], ITIS [42], and Urban Informatics (UI) [43], etc. The car navigation company TomTom [44], has one million GPS users and uses about 80 million other data sources such as GSM to monitor real time traffic and give advice on the best route, taking into account traffic congestion. Google Maps [45] gets live traffic information displaying road way traffic conditions for main roads in many countries updated every five to ten minutes. Close to real-time traffic maps are provided by all larger search companies including Yahoo, Bing, Baidu and Yandex. For example Yandex also provide real time video cameras of selected junctions in Moscow, and traffic map forecast at 15 minutes intervals up to one hour. The Mobile Millennium project at the University of California (UC), Berkeley, in collaboration with Nokia Research Centre, and NAVTEQ Company uses GPS in cellular phones to gather traffic information, process it, and distribute it back to the phones in real time [46].

Microscopic traffic simulation [47] is a field closely related to trajectory mapping. In this context 'microscopic’ means modelling and simulation of the movement of individual vehicles. The vehicles are typically modelled taking into account the distance to the vehicles in front; this allows realistic simulation of congestion waves that move upstream with a group velocity of about 15 *km*/*h*. Several simulation models combining microscopic traffic modelling with simulation of air pollution concentrations in street canyons [48-51] make it feasible to calculate traffic-related air pollution by mapped personal trajectories.

This paper combines the above presented unrelated technologies to form a new approach in urban traffic-related air pollution and public health impact research. We develop a concept for air pollution research based on trajectory mapping by using an empirical model that articulate the overall work process, system architecture, traffic-related air pollution and exposure modelling. We also discuss the main challenges that might be faced by applying such an approach to study the traffic-related air pollution to individual exposure and its public health impact assessment.

## Methods

### Work processes and system architecture

The work process is composed of six parts. Figure 1 illustrates the key elements of the work process. Following the work process in Figure 1, one can both construct the pollution field and estimate the pollution exposure by fixed site data from relatively high density of monitoring stations (steps 1, 5 and 6), or alternatively by mobile phone tracking (steps 2–6), or through combining both mobile phone tracking and fixed site monitoring stations (steps 1–6). The work process is described in detail below.

#### Pollution monitoring using stationary sites

In the suggested approach, the air pollution data can be directly gathered from a static network of pollution monitoring sites across the city and air pollution maps are generated by interpolating between them. People can then check their exposure levels by mapping their individual trajectory into the pollution map. However, there are some limitations of using static monitoring networks, i.e., mainly the total number of air quality monitoring sites within a city is often limited by practical constraints, and possibly other limitations (e.g., reporting hourly averages, possibly not at real time in some regions, etc.). As a possible complementing source of information, high spatial resolution satellite observations can be used. While satellite measurements have many advantages and are developing rapidly, they do have some major limitations, particularly when considering intra-urban resolution. Although most operational satellite products relevant for air quality applications currently do not yet possess the spatial resolution necessary to directly resolve fine spatial detail at the urban scale, their output can nonetheless be very valuable for improving the output of local and regional chemical transport models through data assimilation schemes, and as such indirectly improve urban-scale air quality estimates. Furthermore, research is currently ongoing on producing higher-resolution (300 m) satellite-based PM measurements at the urban scale [52]. Finally, significant progress with regards to spatial resolution achievable from space-based platforms will further be made in the near future with new instruments such as TROPOMI (The TROPOspheric Monitoring Instrument) on the Sentinel-5 precursor platform, which will open up entirely new applications for space-based air quality monitoring, in particular over megacities and other large urban agglomerations.

#### Trajectory mapping by mobile phone

Trajectory mapping uses individual human trajectories generated by advanced mobile phone GSM (GPS-free) tracking systems. The tracking system should be capable of accurately locating mobile phones on road segments in a roadway network by using traffic engineering tracking systems. From frequent location updates, it is possible to track the approximate real-time speed of the mobile phones. This technology is partly qualified, but to date has not been implemented for large populations. Computational capacity may also be a limitation as one PC calculates a one hour individual trajectory in about two minutes [39]. There are ways to use one individual trajectory for calculating other trajectories on the same road segment at approximately the same time to make the computation of a large number of trajectories feasible. Commercial algorithms are likely to be faster on commuter freeway systems where the vehicles, on average, are travelling on a small set of roadways and stay on the same roadway for quite a long time.

#### Automatic vehicle type identification or estimation

A mobile phone signal does not identify a vehicle. The vehicle type can be estimated by drawing randomly or conditioned to trajectories such as bus stop patterns to identify that it is a bus from a national vehicle type distribution. Modern Automatic Vehicle Identification (AVI) systems cover or will soon cover most vehicles in a country such as Autopass [53] in Norway, SINIAV in Brazil [54], and EVR in Mexico [55]. These systems are based on Radio Frequency Identification (RFID) [56] and give the exact date and time for passing a certain check point. Cross correlation of AVI and mobile phone data results in one-to-one assignment of a mobile phone to an identified vehicle. The vehicle type, age, etc., will determine its emission class which is a key to building up a picture of emissions.

#### Vehicle trajectories and vehicle emissions

Individual human trajectories can be further used to generate vehicle trajectories on road segments in a roadway network by using traffic engineering tracking systems. Emissions from each individual vehicle can be calculated along the vehicle trajectories.

#### Pollution field modelling

Traffic pollution is not just a product of vehicle exhaust, but also tyre wear, brakes wear, oil leaks, as well as erosion of the road surface itself and deposited sand and salts at icy/snowy winter roads. Dispersion of traffic air pollution in urban areas can be modelled numerically by taking into account the recirculation effect of street canyons, meteorological conditions, pollution type, traffic counts and types, and other parameters. In this case, various dispersion models (e.g., CALINE3/4 [57] and CAL3QHC/R [58]), CFD (Computational Fluid Dynamics) models [58,59], geo-statistical interpolation models and hybrid models that are specially developed for or simply used in street canyon applications can be applied. To realize this, an increase in computational power is needed, especially if the model is run over long time periods of weeks and even months; and/or to model air pollution level on a street canyon for an entire city could also be computational intensive. Moreover, there is a need for high resolution input data for the models about the meteorology in the city, including temperature and wind speed and directions.

#### Pollution exposure along trajectories

Exposure data can be acquired by evaluating the pollution field along the trajectories in space and time. In this case, a micro-environment based exposure model can be applied [60].

Mobile phone-based mobility tracking used to model traffic pollution contributions to both individual chronic and acute exposure can open new fields of research. This approach can be further developed to estimate the accumulated dose of air pollutants in the human body, and to assess the health impact by coupling individual trajectories to individual physiological information (e.g., age, gender, inhalation rate, body length and weight, health records, etc.) [61,62]. Field studies with personal exposure monitors have found that traditional exposures estimated from average pollution levels in a city based upon residence postal codes are inadequate for pollutants which are spatially variable [16] and tend to underestimate variability in exposure, in particular when a person’s location is highly correlated to narrow peaks in the pollution field. The trajectory based system proposed in this paper will pick up such correlations. Therefore, the individual trajectory approach would be expected to more accurately identify the individuals with the greatest health risk. The most highly exposed groups may share common trajectory patterns, for example those with commuter/work patterns that have the highest health risk (e.g., professional drivers, traffic police and construction workers, etc.). By coupling mobile phones to electronic health records (EHR) and/or personal health records (PHR), one may identify highly vulnerable/pollution-sensitive groups (for example those with respiratory diseases) among the most risky trajectory patterns. Thus, this may open up new perspectives in empirical public health research.

**Figure 2:**
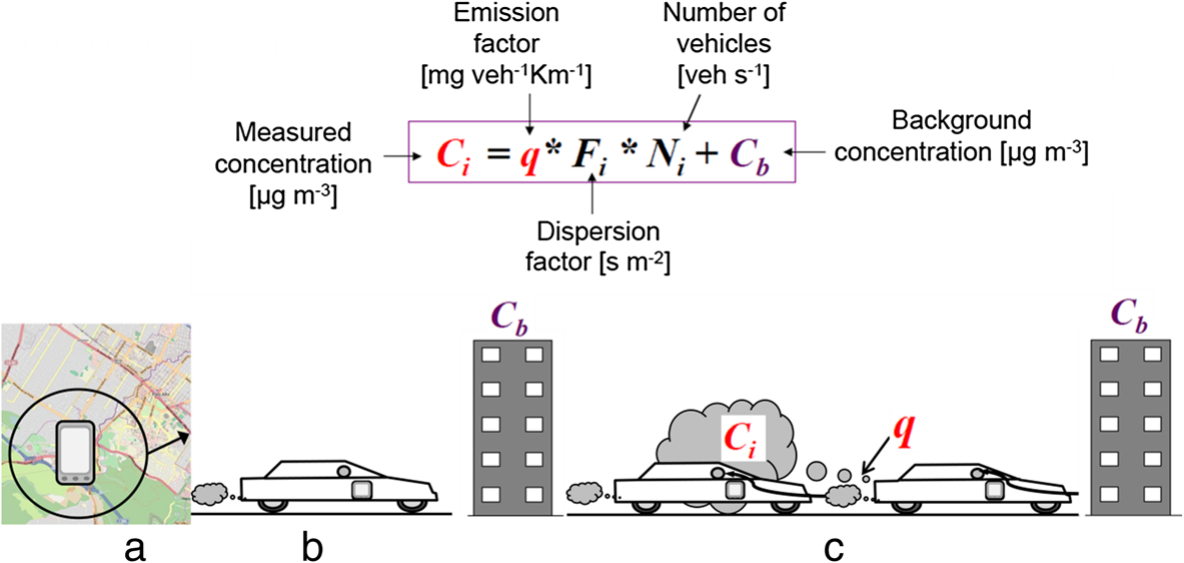
**The potential to characterize traffic-related microenvironments, e.g., in-vehicle exposure in congestion as opposed to non-congestion. (a)** Mapping a mobile phone trajectory; **(b)** Associate mobile phones with vehicles, and associate vehicles to air pollution which enables construction of a continuous air pollution field in space and time; **(c)** Estimate exposure for those people who travelling through the pollution field (the equation is from Reference No. 89).

Figure 2 illustrates the potential to characterize traffic-related microenvironments, e.g., in-vehicle exposure in congestion as opposed to non-congestion: (a) mapping a mobile phone trajectory; (b) associating mobile phone with vehicles, and linking vehicles to air pollution; and (c) estimating exposure for people travelling through the pollution fields [63]. It needs to be noted that the mobile phone is used to identify the person and a person-related vehicle.

**Figure 3:**
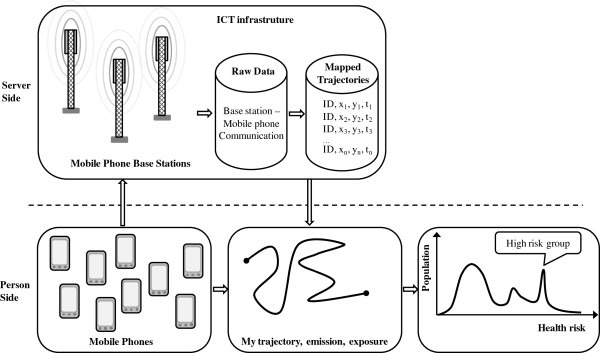
**System architecture for obtaining mapped trajectories and public health impact.** It consists of a two-way communication: server side (mobile phone base stations – raw data – mapped trajectories) and person side (mobile phone – individual trajectory, emission, exposure - health effect).

Figure 3 illustrates the system architecture of a complete system following the work process in Figure 1. On the Information Communication Technology (ICT) infrastructure part, the raw data are the mobile phone base stations in communication with individual mobile phones. These raw data are used to efficiently generate individual mapped trajectories showing the positions of individual mobile phones through time. Mapping algorithms are used to locate the moving positions onto road segments. The mapped trajectories are used as input to algorithms to construct the pollution field and estimate exposure for people moving in the pollution field.

At the population-scale one may investigate high risk groups and check if they share common travel patterns. Note that on the lower right graph in Figure 3 the term 'population’ indicates the population density or frequency in a population histogram. This may be used to identify the population fraction (i.e., area below the curve) and the risk of the high risk group.

### Existing traffic air pollution exposure models

The goal of this section is to review some modelling approaches that can be used in this proposed approach, to calculate the traffic-related air pollution and its contribution to individual exposure. Each given model is kept simple and current at a conceptual level, so that each model can be replaced by more advanced models in specific studies.

#### Dispersion models

A simplifying concept of a roadway air pollution model is to treat the emission from many vehicles as one line source. The pollution is dispersed away from the line source to the entire model domain (and not just near roads) by the mechanisms of diffusion and convection (move with the local velocity of the air). However, due to the dispersive spreading the highest pollution levels are found at the line source. For simpler, roadway-only sources, the CALINE3/4 [58] and CAL3QHC/R [58] series of dispersion models explicitly address traffic-induced emissions. AERMOD is also broadly used for traffic-related complex source mixtures, e.g., terminals, ports and roadways considered in combination, large highway corridors and urban scale modelling [58].

#### Geo-statistical interpolation models

Geo-statistical interpolation models can be implemented to estimate the traffic-related air pollution based on a statistical interpolation of a dense, well-distributed, monitoring work [64]. These models allow the estimation of pollution concentrations over several time intervals, but this is limited only by the number of available measurement periods [64].

#### Hybrid models

The hybrid models are those that combine personal or regional monitoring with other air pollution exposure methods [64]. Most studies were conducted in European cities, and in San Diego [65], e.g., by using personal exposure measurements in conjunction with fixed outdoor stations to estimate hourly exposure for each day, to calculate a traffic-density index [66]; and by using regional monitoring in conjunction with another modelling scheme to assume long-term exposure to NO_2_ and Black Smoke (BS) [67], etc.

### Essential factors to estimate traffic air pollution exposure in the mobile phone tracking approach

No matter which of the above given models is applied, in order to calculate real-time varying traffic-related air pollution, the methods for estimating the traffic emission, emission rates, emission factors, traffic flows, and individual exposure to dynamic traffic air pollution need to be addressed explicitly.

#### Estimation of traffic-related air pollution

The roadway air dispersion models are usually used to estimate traffic-related air pollution. It generally relies on Gaussian plume equation [68], including wind speed, wind direction, traffic wakes and meteorological air stability.

Many tools have been developed to estimate the vehicle emission factors covering a broad range of pollutants and allow multiple scale analysis, such as the U.S. EPA MOVES (Motor Vehicle Emission Simulator) [69] for HC, NOx, CO, gaseous SO2, ammonia, direct PM; the UK Defra (Department of environment, food and rural affairs) EFT (Emission factor toolkit) Version 5.2 for NOx, PM10, PM2.5, CO2 and Hydrocarbons [70]; and the HBEFA (The Handbook Emission Factors for Road Transport) for atmospheric emission factors for regulated and important non-regulated pollutants from road vehicles in Germany, Austria, Switzerland, Sweden, Norway [71,72]; and the EMFAC (EMission FACtors) for HC, CO, NOx, CO2, PM, fuel consumption, oxides of sulphur (SOx), and lead (Pb) developed and used in California [73], etc.

#### Estimation of traffic flows

One challenge in roadway air dispersion modelling is the transformation from a Lagrangian formulation [74] where each vehicle is a particle with a given speed to an Eulerian formulation [75] where each road segment has a vehicle density with a local flow velocity. This particular challenge has been investigated by the UC Berkeley Mobile Millennium project [72] and an Eulerian–Lagrangian cell transmission model for air traffic flow has been developed [76]. In traffic flow conditions, the two most important densities are the critical density (*n*_*c*_) and the traffic jam density (*n*_*jam*_). The maximum density achievable under free flow is *n*_*c*_, while the minimum density achieved under jamming congestion is *n*_*jam*_. In general, traffic jam density is about seven times the critical density. Traffic is, however, a complex phenomenon that is described by physics concepts such as synchronization and self-organized criticality (SOC). Kerner [77] divided traffic flow into three phases from low to high density: 1) free flow, 2) synchronized congested flow, and 3) wide moving jam (wide jam that moves upstream).

The traffic density-interaction between the vehicle wakes (i.e., isolated vehicles, interacting vehicle wakes, strong interacting vehicle wakes) have been studied by Gokhale and Khare [78], and their results showed that in 'unstable’ conditions, the Vehicle Wake Factor (VWF) varies between 1.63 and 0.3 (for wind direction, *θ* = 90°) and 2.5 and 0.8 (for wind direction, *θ* = 180°). During 'neutral’ and 'stable’ conditions, it is in the range of 0.84 – 0.4, 1.91 – 0.85 (for wind direction *θ* = 270°) and 1.7 – 0.7, 3.1 – 0.3 for wind direction *θ* = 360°, respectively.

Not all citizens carry a mobile phone and this makes the mapped population an incomplete set of mapped persons/vehicles. One may fill in these gaps using statistical distributions. For simplicity, we assume 100% mobile phone coverage, that is, each individual has a mobile phone that is tracked. Linking individual trajectories to vehicles can be done as follows: (i) Identify bus drivers and passengers by their characteristic trajectory pattern. They follow a pre-defined route with a given stopping pattern. It is important to correctly estimate the number of persons travelling by bus in order to avoid over estimating the number of vehicles. (ii) Identify private car commuters by their regular trajectory patterns from the suburb to the city, and *vice versa*. (iii) Identify other group travellers (e.g., subway commuters, pedestrians, motorcycles, bicycles riders) by having an identical trajectory. This is to avoid overestimating the number of vehicles with passengers. (iv) The rest of the trajectories are individual drivers and their vehicles may be stochastically drawn from the vehicle type (e.g., electric car, truck or lorry) including the vehicle age, probability density function.

The road network is discretized into piecewise linear road segments. The pollution from each segment is modelled by the linear source equation. The pollutant concentration for people on the road segment is given by the characteristic mid-segment pollution. The vehicle densities (number per length) are estimated by the procedure above. The average speed of traffic in the segment is calculated by the trajectory data (∆X,∆Y,∆t) and ∆Z from terrain/map data.

If the road is a freeway with many lanes one may consider splitting the two driving directions into two line sources and sum the pollution from the two directions taking into account the centre-to-centre distance between the lane directions. For near roadway exposure one may select bands parallel to the roadway network and take into account overlapping contributions from the nearest neighbouring road(s). Traffic also contributes to background pollution, and this may be calculated by longer term smoothing of the total city pollution from stationary sites’ data.

#### To model individual exposure to traffic air pollution

Human exposure refers to an individual’s contact with a pollutant concentration. Given a close to real-time continuous pollution field, it is straightforward to calculate the direct exposure to air pollution in the transport network. In this context, a microenvironment (e.g., the interior of a car/bus, inside a house, or urban, suburban, and rural areas, etc.) based integrated exposure model can be applied and expressed as [60]:

$$X_{i}=\sum_{j}^{J}C_{j}t_{\mathrm{ij}}$$

Where *X*_*i*_ is the total exposure for person *i* over the specified period of time, *C*_*j*_ is the pollutant concentration in microenvironment *j*, *t*_*ij*_ is the residence time of the person *i* in microenvironment *j*, and *J* is the total number of microenvironments. The total exposure is, therefore, the sum of exposures during a given time and has thus the same dimension as exposure in general: (mass) × (time) / (volume) or (concentration) × (time), for example, with units *μg d m*^*–3*^.

In the case where *M* persons are exposed to the same concentration for the same time, the exposure in this case is *M* times that for one person and, for example, with the units (concentration) × (time). The resulting population exposure may then be used to relate the number of hospital admissions to exposure.

A constraint for using the microenvironment based exposure models (see equation above) is that the residence time of the person (termed the time–activity pattern) needs to be known together with the pollution concentrations in each of the microenvironments at the time when the person is present. In this article, however, by using mobile phone tracking we could get both individual time-activity pattern and real time continuous pollution concentration along the traffic network.

## Discussion

Two alternatives to an individual trajectory based system are: (1) one may measure real-time air pollution by large-scale implementation of a Wireless Sensor Network (WSN) [79-82], in which hundreds or thousands of small personal environmental sensors carried by the public rely on cell phones to shuttle information to central computers where it will be analysed, made anonymous and reflected back to individuals, authorities and public health agencies [83]; and (2) one may rely on stationary based, monitored pollution data together with geo-statistical models and use GPS information [84-86] provided by smart phone applications to track air pollution exposure of a sub-population [87-89] as mentioned earlier in this article. Alternative (1) uses individual trajectories. One may expect that an actual measurement of the air pollution exposure by WSN would be somewhat better than a calculated individual trajectory based system as proposed in this article. This is a point in favour of the WSN-based micro sensor approach. However, the cost of equipping a population with environmental micro sensors limits this method to small-scale studies (Table 1). Alternative (2) for an entire city would require stationary measurements at nearly all road segments in the city. Although this could provide high quality data, it is certainly extremely costly due to the extremely high density of measurement stations. It scales with the size of the road network as shown in Table 1. The cost of up-scaling a mobile phone trajectory system is only the cost of incremental computational power. This is very low since the individual data already exists and can be uploaded from the mobile phone or downloaded from the mobile phone service provider. Less computational challenging studies have already included six million phone users of a Call Detailed Records (CDR) data base [90]. The detailed traffic trajectories need more computational power, but the algorithms are cost-efficient and are likely very well suited for parallel algorithms, since each trajectory is calculated independently of the other trajectories. Trajectory based data have many opportunities for reducing costs by building on synergies with commercial traffic monitoring systems. A commercial traffic monitoring system may add a health impact service that not only optimizes commuting routes based on the time spent from home to work, but may also propose alternative eco-driving routes that have a positive impact on one’s personal health.

**Table 1:** Comparison of alternatives to the base case proposed in this article

**Case**	**Pollution field**	**Exposure**	**Cost at population scale**
Base case in this article	Pollution field is calculated by the aggregate of individual GSM or GPS-based trajectories plus emission and dispersion models.	Accumulated along individual GSM or GPS-based trajectories.	Low cost per individual. Cost of computational power.
Alternative 1	Measured on each individual trajectory by people carrying environmental micro-sensors.	Accumulated along individual GPS-based trajectories.	High cost per individual. Additional cost per individual is roughly the cost of a new smart phone.
Alternative 2	Stationary monitoring network plus geo-statistics modelling.	Accumulated along GSM or GPS-based trajectories.	Very high investment cost. Need very high density of monitoring stations on the road network.

Traffic monitoring companies, including the largest search engine companies, may want to show their social responsibility by releasing their data for public health research in the same way as mobile phone tracking data are released for human mobility research. In this way the public health research could be obtained at almost no extra cost. More realistic, we believe that traffic monitoring companies will offer new free services to calculate real time pollution exposure and health risk maps in large cities that can be used by traffic map users with GPS smart phones. We have developed a model to estimate traffic emissions from traffic map data. This modelling is planned to be published in another article. Based on this approach a mobile phone tracking system has the potential of being highly competitive economically for population-wide traffic pollution health studies.

Trajectory based individual exposure analysis opens up new perspectives in public health research. Instead of analysing either the average health risk given by average city pollution levels or analysing a limited number of citizens’ mobility, we are now in a position to analyse the probability distribution density of individual risk for relevant diseases. Furthermore, by linking to HER and/or PHR, the proposed approach can help to better understand the impact of environmental exposure on human health at both individual and population levels, and might enhance environmental public health tracking as well [91-93]. Natural questions that arise include: Who is most at health risk? Is it the large majority of the population with moderate pollution exposure or a small group of highly sensitive/vulnerable persons with unusual exposure trajectories? A natural extension of the trajectory based individual exposure analysis is to do research on optimal regulatory, policy change and urban planning to reduce air pollution. What are the most efficient ways to transform an existing urban environment into a city with a low air pollution health risk?

In polluted cities there are many different options for improving air quality. One may develop trajectory based dynamic computer simulation models where urban planning concepts are evaluated based on simulated performance with regard to cost and improved public health. This approach is similar to dynamic petroleum reservoir simulation by streamlines [94].

A vision for mobile phone tracking technology is to do global studies that incorporate the global gaps in population of mobile phone users. The gaps in the network, that is, those that do not have a mobile phone, may be inferred directly from the trajectories from home to kindergarten, to school, to work, and scale this number of trajectories to estimate the effect from the whole population. An advantage of this approach is that it is effortless for citizens as the global population does not need to adjust their activity pattern or wear any measurement devices to participate in the study.

There are some limitations to our approach that need to be addressed when used in real practice. First, the proposed approach is mainly targeted at the person who has a mobile phone. This is a limitation for those people that do not tend to have mobile phones (e.g., children, elderly, the poor, etc.). The biased samples are not easily worked out with statistical methods if they are not randomly distributed, which is the case here. Second, the risks arise first from the fact that in order to get the individual human trajectory, the data we need is mostly about people, where they have been, at what times, how often. Therefore privacy and security is a major concern, and needs to be addressed before the opportunities of trajectory mapping can be created. Furthermore, there are also huge issues with privacy and access to link to HER and/or PHR. Encryption and anonymization can provide a level of privacy [95]. In this study, anonymous data are enough to construct individual human trajectories, pertaining to groups of people. However, to guarantee anonymity is hard in the domain to link/compare the spatio-temporal individual trajectory based exposure-dose modelling with real medical patient data. Many studies that address privacy issues in a spatio-temporal data mining context have already been published. In the Geographic Privacy-Aware Knowledge Discovery and Delivery (GeoPKDD) project, Giannotti and Pedreschi [96] investigated the various scientific and technological issues of mobility data, open problems, roadmap, and concluded that the privacy issues related to the ICTs can only be addressed through an alliance of technology, legal regulations and social norms. In the meantime, increasingly sophisticated privacy-preserving data mining techniques are being studied and further developed [97]. Their aim is to achieve appropriate levels of anonymity by means of controlled transformation of data and/or patterns – limited distortion that avoids the undesired side effect on privacy while preserving the possibility of discovering useful knowledge. The project Citisense designed a permissioning system that allows users to configure publicly accessible time Windows of their data [98]. Mun et al. [18] suggested a selective sharing and hiding mechanism in PEIR that enable users to share their location traces with people they trust, and generates believable proxy traces for times a user does not want their real location revealed. Furthermore, an alternative to deal with privacy issues related to tracking of individual trajectories is to collect a population-wide data in the form of willing participation, as done by PEIR [17,18] mentioned above, where only individuals that are motivated will take part in the data collection. In this approach, the methods to empower citizens at population level to participate in such studies may need to be further developed.

Another limitation of using mobile phone tracking approach to study individual exposure to traffic-related air pollution and its public health impact assessment is that the external data/information and models needed, such as vehicle type identification, emission dispersion models, emission factors and meteorology data needs to be known together with the vehicle trajectories to model pollution fields; individual physiological information (e.g., height, weight, inhalation rate) is needed in each of the microenvironments at the time when the person is present in order to estimate the accumulated dose in the human body, etc. So this approach also suffers from similar data problems to the more conventional dispersion models. On the other hand, such challenges may indicate that the approach of modelling traffic-related air pollution and its implications for public health impact assessment through mobile phone tracking will require broad cooperation within inter- and multidisciplinary disciplines.

## Conclusion

We conclude that there is an opportunity to calculate traffic air pollution and its public health impact by mobile phone trajectory mapping. This approach is promising due to the following characteristics: (i) **Low cost**: There is no cost from the observation part. The cost is mainly in data power to compute mobile phone trajectories and emission dispersion modelling in real time. Combination of low cost and standardized mobile phone data makes it is feasible to do population-wide traffic pollution studies. The system is suited for developing countries that have a low fraction of mobile phones with GPS. (ii) **Near real-time**: The system is based on fully automatic calculation and real-time data. One may obtain real-time pollution/exposure fields. This allows identification of high vehicle density congestion events that may contribute significantly to health risk (iii) **Effortless citizen participation**: Each trajectory reflects one individual citizen. Therefore, this system opens up the possibility of providing information to each individual citizen. One may develop applications for automatic web server feedback to citizens who want to get the data of their own trajectories or want to contribute toward a training data set of true ground GPS measurements. We believe that using the trajectories of individual mobile phones opens up new perspectives on urban dynamics and public health research.

## Abbreviations

AOA: Angle of arrival; AOD: Aerosol optical depth; AVI: Automatic vehicle identification; CDR: Call detailed records; CitiSense: Adaptive services for community-driven behavioural and environmental monitoring to induce change; CO: Carbon oxides; EFT: Emission factors toolkit; EHR: Electronic health records; EMFAC: EMission FACtors; GASP: GEOS aerosol/smoke product; GE: The general electric company; GeoPKDD: Geographic privacy-aware knowledge discovery and delivery; GOES: Geostationary operational environmental satellite; GPS: The global positioning system; GSM: The global system for mobile communications; HBEFA: The handbook emission factors for road transport; HC: Hydrogen carbon; HMM: Hidden markov model; ICT: Information communication technology; IMS: Intelligent mechatronic systems inc; LUR: Land use regression; LBS: Location-based services; MIT: The massachusetts institute of technology; MODIS: Moderate resolution imaging spectro-radiometer; MOVES: Motor vehicle emission simulator; NO2: Nitrogen dioxides; NOx: Nitrogen oxides; OMI: Ozone monitoring instrument; Pb: Lead; PEIR: The personal environmental impact report; PHR: Personal health records; PM: Particulate matter; RFID: Radio frequency identification; SO2: Sulphur dioxides; SOx: Oxides of sulphur; TDOA: Time difference of arrival; TOA: Time of arrival; TROPOMI: The TROPOspheric Monitoring Instrument; TSP: Total suspended particulates; UC: University of California; UI: Urban informatics; UN: The United Nations; USC: The University of Southern California; U.S. EPA: The United State environmental protection agency; VWF: Vehicle wake factor; WHO: The World Health Organization; WSN: Wireless sensor network.

## Competing interests

The authors declare that they have no competing interests.

## Authors’ contributions

HYL and ES planed this work and wrote the manuscript. MJK contributed content and provided editorial input. All authors approved the final version.

## References

[B1] UN report World’s biggest cities merging into 'mega-regions’ [http://www.guardian.co.uk/world/2010/mar/22/un-cities-mega-regions]

[B2] HEI Panel on the Health Effects of Traffic-Related Air Pollution Traffic-related air pollution: a critical review of the literature on emissions, exposure, and health effects 2010 Boston, Massachusetts: Special Report 17

[B3] Padmanabhan N Glenn B EPA research focus – health effects of near-roadway air pollution [http://pubs.awma.org/gsearch/em/2009/8/padmanabhan.pdf]

[B4] Fruin S Westerdahl D Sax T Sioutas C Fine PM Measurements and predictors of on-road ultrafine particle concentrations and associated pollutants in Los Angeles Atmos Environ 2008 42 207 219 10.1016/j.atmosenv.2007.09.057

[B5] Rank J Folke J Jespersen PH Differences in cyclists and car drivers’ exposure to air pollution from traffic in the city of Copenhagen Sci Total Environ 2001 279 131 136 10.1016/S0048-9697(01)00758-6 11712590

[B6] van Wijnen JH Verhoeff AP Jans HW van Bruggen M The exposure of cyclists, car drivers and pedestrians to traffic-related air pollutants Int Arch Occup Environ Health 1995 67 187 93 7591177 10.1007/BF00626351

[B7] Soll-Johanning H Bach E Olsen JH Tuchsen F Cancer incidence in urban bus drivers and tramway employees: a retrospective cohort study Occup Environ Med 1998 55 594 598 10.1136/oem.55.9.594 9861180 PMC1757639

[B8] WHO More die from car pollution than road accidents [http://www.ibike.org/environment/air-pollution.htm]

[B9] Pope CA Dockery DW Health effects of fine particulate air pollution: lines that connect J Air Waste Manage Assoc 2006 56 709 742 10.1080/10473289.2006.10464485 16805397

[B10] Halonen JI Lanki T Yli-Tuomi T Kulmala M Tiittanen P Pekkanen J Urban air pollution and asthma and COPD hospital emergency room visits Thorax 2008 63 574 476 10.1136/thx.2008.096073 18267984

[B11] Sørensen M Hoffmann B Hvidberg M Ketzel M Jensen SS Andersen ZJ Tjønneland A Overvad K Raaschou-Nielsen O Long-term exposure to traffic-related air pollution associated with blood pressure and self-reported hypertension in a Danish cohort Environ Health Perspect 2012 120 418 24 10.1289/ehp.1103631 22214647 PMC3295339

[B12] Raducan GM Pollutant dispersion modelling with OSPM in a street canyon from Bucharest Romanian Report in Physics 2008 60 1099 1114

[B13] Clougherty JE Wright RJ Baxter LK Levy JI Land use regression modelling of intra-urban residential variability in multiple traffic-related air pollutants Environ Health 2008 7 17 10.1186/1476-069X-7-17 18485201 PMC2397396

[B14] Henderson SB Beckerman B Jerrett M Brauer M Application of land use regression to estimate long-term concentrations of traffic-related nitrogen oxides and fine particulate matter Environ Sci Technol 2007 41 2422 2428 10.1021/es0606780 17438795

[B15] Marshall JD Nethery E Brauer M Within-urban variability in ambient air pollution: comparison of estimation methods Atmos Environ 2008 42 1359 1369 10.1016/j.atmosenv.2007.08.012

[B16] Dijkema M Traffic Related Air Pollution Spatial Variation, Health Effects and Mitigation Measures PhD thesis 2011 Utrecht University: Institute for Risk Assessment Sciences

[B17] UCLA CENS PEIR: personal environmental impact report [http://www.viewingspace.com/peir.html]

[B18] Mun M Reddy S Shilton K Yau N Burke J Estrin D Hansen M Howard E West R Boda P Zielinski K, Wolisz A, Flinn J, LaMarca A PEIR, the personal environmental impact report, as a platform for participatory sensing systems research Proceedings of MobiSys 2009: the 7th international conference on mobile systems, applications and services: 22–25, June, 2009; Wroclaw, Poland 2009 New York, USA: ACM 55 68

[B19] Reddy S Mun M Burke J Estrin D Hansen M Srivastava M Using mobile phones to determine transportation modes ACM Transactions on Sensor Networks (TOSN) 2010 6 13 1 13

[B20] Stenneth L Wolfson O Yu PS Xu B Agrawal D, Cruz I, Jensen CS, Ofek E, Tanin E Transportation mode detection using mobile phones and GIS information Proceedings of GIS '11: the 19th ACM SIGSPATIAl international conference on advances in geographic information systems: 1–4, November, 2011; Chicago, IL, USA 2011 New York, USA: ACM 54 63

[B21] ENVIROFI: The environmental observation web and its service applications within the future internet [http://www.envirofi.eu/]

[B22] CitiSense Adaptive services for community-driven behavioural and environmental monitoring to induce change [https://sosa.ucsd.edu/confluence/display/CitiSensePublic/CitiSense]

[B23] Pau G Tse R Challenges and opportunities in immersive vehicular sensing: lessons from urban deployments Signal Process Image Commun 2012 27 900 908 10.1016/j.image.2012.01.015

[B24] Bales E Nikzad N Quick N Ziftci C Patrick K Griswold W Chlamtac I, Mayora-Ibarra O, Varshney U, Osmani V Citisense: mobile air quality sensing for individuals and communities design and deployment of the citisense mobile air-quality system Proceedings of 6th international conference on pervasive computing technologies for healthcare (Pervasive Health): 21–24, May, 2012; San Diego, California, USA 2012 New York: IEEE Xplore Digital Library 155 158

[B25] Yeung CH Saad D Networking – a statistical physics perspective J Phys A Math Theor 2013 46 103001 10.1088/1751-8113/46/10/103001

[B26] Paul U Subramanian AP Buddhikot MM Das SR Gopalan K, Striegel AD Understanding traffic dynamics in cellular data networks Proceedings of INFOCOM: 10–15 April 2011; Shanghai, P.R. China 2011: IEEE Xplore Digital Librry 882 890

[B27] Barabási A-L Albert R Emergence of scaling in random networks Science 1999 286 509 512 10.1126/science.286.5439.509 10521342

[B28] Barabási A-L Albert R Statistical mechanics of complex networks Rev Mod Phys 2002 74 47 97 10.1103/RevModPhys.74.47

[B29] Song C Koren T Wang P Barabási A-L Modelling the scaling properties of human mobility Nat Phys 2010 10 818 823

[B30] Wang D Pedreschi D Song C Giannotti F Barabasi AL Chid A, Joydeep G, Padhraic S Human mobility, social ties, and link prediction Proceedings of KDD 11, the 17th ACM SIGKDD international conference on Knowledge discovery and data mining: 21–24 August 2011; San Diego, California, USA 2011: ACM Digital Library 1100 1108

[B31] Bagrow JP Lin Y-R Mesoscopic structure and social aspects of human mobility PLoS One 2012 7 1 6 10.1371/journal.pone.0037676 PMC3365118 22701529

[B32] Girardin F Vaccari A Gerber A Biderman A Ratti C Yeh AGO, Zhang F Towards estimating the presence of visitors from the aggregate mobile phone network activity they generate Proceedings of the 11th international conference on computers in urban planning and urban management (CUPUM2009): 16–18 June 2009; Hong Kong, P.R. China 2009 Hong Kong: University of Hong Kong 1 11

[B33] Wang S Min J Yi BK Location based services for mobiles i: technologies and standards [http://blue-penguin.org/cache/location-based-services-for-mobiles.pdf]

[B34] Wang G van den Bosch FHM Kuffer M Modelling urban traffic air pollution dispersion Int Arch Photogramm Remote Sens Spat Inf Sci 2008 XXXVII 153 158

[B35] Thiragarajan A Biagioni J Gerlich T Eriksson J Beutel J, Ganesan D, Stankovic J Cooperative Transit Tracking Using GPS-enabled Smart-phones Proceedings of the 8th ACM conference on embedded networked sensor systems: 3–5 November 2010; Zurich, Switzerland 2010 New York: ACM 85 98

[B36] Thiragarajan A Madden S Wang JTL Querying continuous functions in a database system Proceedings of the ACM SIGMOD international conference on management of data, SIGMOD 2008: 10–12 June 2008; Vancouver, BC, Canada 2008 New York: ACM 791 804

[B37] Thiragarajan A Ravindranath L LaCurts K Madden S Balakrishnan H Toledo S Eriksson J USENIX Association Accurate, low-energy trajectory mapping for mobile devices NSDI’11Proceedings of 8th USENIX Symposium on Networked Systems Design and Implementation (NSDI) 2011 Boston, MA, USA: Berkeley: USENIX Association

[B38] Thiragarajan A Ravindranath L LaCurts K Toledo S Eriksson J Madden S David E, Culler , Jie L, Matt W VTrack: accurate, energy-aware road traffic delay estimation using mobile phones Proceedings of the 7th international conference on embedded networked sensor systems, SenSys 2009: 4–6 November 2009; Berkeley, California, USA 2009 New York: ACM 85 98

[B39] Thiragarajan A Probabilistic models for mobile phone trajectory estimation Dissertation 2011: Massachusetts Institute of Technology, Department of Electrical Engineering and Computer Science

[B40] Cellint Cellient traffic solutions making it work for you [http://www.cellint.com]

[B41] IMS Connected car solutions for life [http://www.intellimec.com]

[B42] ITIS Integrated transport information system [http://www.itis.com.my/atis/index.jsf]

[B43] UI Urban informatics [http://www.uinformatics.com]

[B44] TOMTOM [http://www.tomtom.com/en_gb/localechange.jsp]

[B45] Google Maps [https://maps.google.com/]

[B46] Herrera JC Work D Ban X Herring R Jacobson Q Bayen A Evaluation of traffic data obtained via GPS-enabled mobile phones: the mobile century field experiment Transportation Research C 2010 18 568 583 10.1016/j.trc.2009.10.006

[B47] Treibner M Kresting A An open source microscopic traffic simulator IEEE Intell Transp Syst Mag 2010 2 6 13

[B48] Khalesian M Pahlavani P Delavar MR GIS-based multi-agent traffic micro simulation for modelling the local air pollution Int Arch Photogramm Remote Sens Spat Inf Sci 2008 XXXVII 491 496

[B49] López-Neri E Ramírez-Treviño A López-Mellado E A modelling framework for urban traffic systems microscopic simulation Simul Model Pract Theory 2010 18 1145 1161 10.1016/j.simpat.2009.09.007

[B50] Nökel K Schimdt M van Vuren T Meersman H, van de Voorde E, Winkelmans W An integrated dynamic traffic simulation and air pollution decision support system Proceedings of 8th world conference on transport research; volume 2: planning, operation, management and control: 12–17 July 1998; Antwerp, Belgium 1999 Victoria: ARRB Group Limited 237 250

[B51] Vardoulaki S Fisher BEA Pericleous K Gonzalez-Flesca N Modelling air quality in street canyons: a review Atmos Environ 2003 37 155 182 10.1016/S1352-2310(02)00857-9

[B52] Glantz P Kokhanovsky A von Hoyningen-Huene W Johansson C Estimating PM2.5 over southern Sweden using space-borne optical measurements Atmos Environ 2009 43 36 5838 5846 10.1016/j.atmosenv.2009.05.017

[B53] AutoPASS [http://www.autopass.no]

[B54] Siniav [http://siniav.net]

[B55] EVR [http://www.electronicvehicleregistration.com]

[B56] RFID [http://en.wikipedia.org/wiki/Radio-frequency_identification]

[B57] Bailey C Dispersion modelling for mobile source applications [http://www.cleanairinfo.com/regionalstatelocalmodelingworkshop/archive/2009/presentations/06%20Thurs%20AM/Bailey_Dispersion%20Modeling%20for%20Mobile%20Source%20Applications.pdf]

[B58] Wang YJ Zhang KM Modelling near-road air quality using a computational fluid dynamics model, CFD-VIT-RIT Environ Sci Technol 2009 43 7778 7783 10.1021/es9014844 19921893

[B59] Vardoulakis S Fisher BEA Pericleous K Gonzalez-Flesca N Modelling air quality in street canyons: a review Atmos Environ 2003 37 155 182 10.1016/S1352-2310(02)00857-9

[B60] Time micro-environment activity modeling [http://www.integrated-assessment.eu/node/204]

[B61] U.S. EPA Exposure factors handbook 2011 edition (Final) 2011 Washington, DC:

[B62] U.S. EPA report Extrapolation of the benzene inhalation unit risk estimate to the oral route of exposure http://www.epa.gov/iris/supdocs/benzsup.pdf.

[B63] Belalcazar LC Clappier A An alternative technique to estimate road traffic emissions [http://www.epa.gov/ttnchie1/conference/ei20/session7/lbelalcazar_pres.pdf]

[B64] Jerrett M Arain A Kanaroglou P Beckerman B Potoglou D Sahsuvaroglu T Morrison J Giovis C A review and evaluation of intraurban air pollution exposure models J Expo Anal Environ Epidemiol 2005 15 185 204 10.1038/sj.jea.7500388 15292906

[B65] Liu LJ Delfino R Koutrakis P Ozone exposure assessment in a southern California community Environ Health Perspect 1997 105 58 65 10.1289/ehp.9710558 9074882 PMC1469853

[B66] Zmirou D Gauvin S Pin I Five epidemiological studies on transport and asthma: objectives, design and descriptive results J Expos Anal Environ Epidemiol 2002 12 186 196 10.1038/sj.jea.7500217 12032815

[B67] Hoek G Fischer P Van Den BP Goldbohm S Brunekreef B Estimation of long-term average exposure to outdoor air pollution for a cohort study on mortality J Expos Anal Environ Epidemiol 2001 11 459 469 10.1038/sj.jea.7500189 11791163

[B68] Bellander T Berglind N Gustavsson P Jonson T Nyberg F Pershagen G Järup L Using geographic information systems to assess individual historical exposure to air pollution from traffic and house heating in Stockholm Environmental Health Perspective 2001 109 633 639 10.1289/ehp.01109633 PMC1240347 11445519

[B69] U.S. EPA MOVES (Motor Vehicle Emission Simulator) [http://www.epa.gov/otaq/models/moves/index.htm] 10.1080/10962247.2015.1025150 26079557

[B70] Defra Emissions [http://laqm.defra.gov.uk/review-and-assessment/tools/emissions.html#eft]

[B71] IEHIAS emission factors [http://www.integrated-assessment.eu/guidebook/emission_factors]

[B72] Handbook emission factor for road transport (HEEFA) http://www.hbefa.net/e/index.html

[B73] California Air Resources Board EMFAC software [http://www.dot.ca.gov/hq/env/air/pages/emfac.htm]

[B74] Park JY Microscopic modelling of air pollution from road traffic PhD thesis 2005 Imperial College London: University of London Centre for Transport Studies

[B75] Wang Y Zhang KM Modelling near-road air quality using computational fluid dynamics (CFD) model ES&T 2009 43 7778 7783 10.1021/es9014844 19921893

[B76] Bartonova A Clench-Aas J Gram F Gronskei KE Guerreiro C Larssen S Tønnesen DA Walker SE Air pollution exposure monitoring and estimation: Part V. Traffic exposure in adults J Environ Monit 1999 1 337 340 10.1039/a902780g 11529132

[B77] Kerner BS Introduction to modern traffic flow theory and control 2009 Springer: The Long Road to Three-Phase Traffic Theory

[B78] Gokhale S Khare M Vehicle wake factor for heterogeneous traffic in urban environments Int J Environ Pollut 2007 30 97 105 10.1504/IJEP.2007.014505

[B79] Ma Y Richards M Ghanem M Guo Y Hassard J Air pollution monitoring and mining based on sensor grid in London Sensors 2008 8 3601 3623 10.3390/s8063601 PMC3714656 27879895

[B80] Khedo KK Perseedoss R Mungur A A wireless sensor network air pollution monitoring system Int J Wireless Mobile Network 2010 2 31 45

[B81] Mishra SA Tijare DS Asutkar GM Design of energy aware air pollution monitoring system using WSW Int J Adv Eng Tech 2011 1 107 116

[B82] Demin Wang D Agrawal DP Toruksa W Chaiwatpongsakorn C Lu M Keener TC Monitoring ambient air quality with carbon monoxide sensor-based wireless network Commun ACM 2010 53 138 141

[B83] Tracking Air Pollution by Cell Phone [http://pollutionfree.wordpress.com/2009/12/15/tracking-air-pollution-by-cell-phone/]

[B84] Schreiner C Branzila M Trandabat A Ciobanu RC Air quality and pollution mapping system, using remote measurements and GPS technology Global NEST Journal 2006 8 315 323

[B85] Trandabăţ A Branzila M Donciu C Pîslaru M Ciobanu RC Using GPS technology and distributed measurement system for air quality mapping of residential area Environ Eng Manag J 2007 6 545 548

[B86] Al-Ali AR A mobile GPRS-sensors array for air pollution monitoring Sensors Journal, IEEE 2010 10 1666 1671

[B87] Sherwood CH Using GPS and a smart phone to track air pollution exposure [http://www.smartplanet.com/blog/pure-genius/using-gps-and-a-smartphone-to-track-air-pollution-exposure/5578]

[B88] Science Daily Smartphone app measures particulate air pollution [http://www.sciencedaily.com/releases/2010/09/100920135137.htm]

[B89] Bayir MA Demirbas M Cosar A A web-based personalized mobility service for smartphone applications Comput J 2011 54 800 814 10.1093/comjnl/bxq027

[B90] Wang D Pedreschi D Song C Giannotti F Barabasi AL Apte C, Ghosh J, Smyth P Human mobility, social ties, and link prediction Proceedings of KDD '11 the 17th ACM SIGKDD international conference on Knowledge discovery and data mining: 21–24 August 2011; San Diego, California, USA 2011 New York: ACM 1100 1108

[B91] Fitzgerald E Wartenberg D Thompson WD Houston A Birth and fetal death records and environmental exposures: promising data elements for environmental public health tracking of reproductive outcomes Public Health Rep 2009 124 6 825 830 19894425 10.1177/003335490912400610 PMC2773946

[B92] Environmental Exposure Modules for EHR and PHR Systems [http://www.hoise.com/vmw/10/articles/vmw/LV-VM-08-10-6.html]

[B93] Environmental Exposure & EHR Systems [http://www.openhealthnews.com/blogs/groenpj/2011-04-11/environmental-exposure-ehr-systems]

[B94] Fanchi J Principles of Applied Reservoir Simulation 2006 3 Burlington: Elsevier GPP

[B95] Nikzad N Verma N Ziftci C Raij A, Venkatasubramanian K CitiSense: Improving Geospatial Environmental Assessment of Air Quality Using a Wireless Personal Exposure Monitoring System Proceedings of Wireless Health: 23-25 October 2012; San Diego, Califonia, USA 2012 New York: ACM 1 8

[B96] Giannotti F Pedreschi D Giannotti F, Pedreschi D Mobility, data mining and privacy: a vision of convergence Mobility, data mining and privacy: geographic knowledge discovery 2008 New York: Springer 1 14

[B97] Bonchi F Saygin Y Verykios VS Atzori M Gkoulalas-Divanis A Kaya SV Savas E Giannotti F, Pedreschi D Privacy in Spatiotemporal Data Mining Mobility, data mining and privacy: geographic knowledge discovery 2010 New York: Springer 297 334

[B98] Ziftci C Nikzad N Verma N Leavens GT Citisense: mobile air quality sensing for individuals and communities Proceedings of the third annual conference on systems, programming, and applications: software for humanity (SPLASH’12): 19-26 October 2012;Tucson, Arizona 2012 New York: ACM Digital Library 23 24

